# Multi-Class Classification of Breast Ultrasound Images Using Vision Transformer-Based Ensemble Learning

**DOI:** 10.3390/diagnostics15172235

**Published:** 2025-09-03

**Authors:** Tuğçe Taşar Yıldırım, Orhan Yaman, İrfan Kılıç, Beyda Taşar, Esra Suay Timurkaan, Nesibe Aydoğdu

**Affiliations:** 1 Department of Internal Medicine, Fethi Sekin City Hospital, Elazig 23300, Türkiye; dresrasuay@gmail.com (E.S.T.); nesibeaydogdu@gmail.com (N.A.); 2 Department of Digital Forensics Engineering, Firat University, Elazig 23119, Türkiye; orhanyaman@firat.edu.tr; 3 Faculty of Engineering, Department of Software Engineering, Firat University, Elazig 23119, Türkiye; irfankilic@firat.edu.tr; 4 Faculty of Engineering, Department of Mechatronics Engineering, Firat University, Elazig 23119, Türkiye; btasar@firat.edu.tr

**Keywords:** ensemble learning, focal loss, medical image classification, region of interest (ROI) segmentation, vision transformer, breast ultrasound images

## Abstract

**Background/Objectives**: In this study, a vision transformer (ViT) based ensemble architecture was developed for the classification of normal, benign, and malignant diseases from breast ultrasound images. The breast ultrasound images (BUSI) dataset was used for the implementation of the proposed method. This dataset includes 133 normal, 437 benign, and 210 malignant ultrasound images. **Methods**: ROI segmentation and image preprocessing were applied to the dataset to select only the tumor region and use it in the model. Thus, a better performance was achieved using only the lesion regions. Image augmentation was performed using the Albumentations library to increase the number of images. Feature extraction was performed on the obtained images using three ViT-based models (ViT-Base, DeiT, ViT-Small). The purpose of using three different models is to achieve high accuracy. The extracted features were classified using a multilayer perceptron (MLP). Training was performed using 10-fold stratified cross-validation. **Results:** The purpose of stratified cross-validation is to include a certain number of images from all three classes in each cross-validation proposed model provided 96.2% precision and 86.3% recall for the benign class and 92.9% recall and 76.4% precision for the malignant class. The normal class achieved 100% success. The area under the curve (AUC) values were 0.97, 0.96, and 1.00 for benign and malignant tumors, respectively, and 1.00 for normal tumors. **Conclusions:** The ROI-based ViT + MLP + Ensemble architecture provided higher accuracy and explainability compared to traditional convolutional neural network (CNN) based methods in medical image classification. It demonstrated a stable success, especially in minority classes, and presented a potential, reliable, and flexible solution in clinical decision support systems.

## 1. Introduction

Breast cancer is one of the most common types of cancer in women. The mortality rates due to breast cancer are high worldwide [1]. Millions of women are diagnosed with breast cancer every year. Mortality rates of this disease are directly related to the time of diagnosis. Mortality rates can be reduced with an early breast cancer diagnosis and treatment. Therefore, the development of cancer screening, examination, and imaging technologies is extremely important [2,3]. According to World Health Organization (WHO) data, in 2022, 670,000 people died due to breast cancer [4]. According to research conducted worldwide, breast cancer is the most common type of cancer in women in 157 of 185 countries. Breast cancer is a type of disease seen in every country worldwide [4]. The World Health Organization (WHO) aims to prevent 2.5 million deaths from breast cancer between 2020 and 2040 by reducing global breast cancer mortality rates by 2.5% per year. To reduce the breast cancer mortality rate worldwide and prevent 25% of deaths by 2030 and 40% by 2040. To achieve these goals, regular health check-ups, early diagnosis, and comprehensive breast cancer management are required [4]. Early diagnosis methods are usually performed with medical imaging techniques such as mammography, magnetic resonance imaging (MRI) [5], and ultrasound (US) [2,6,7,8,9]. Early diagnosis methods include medical imaging techniques such as mammography, magnetic resonance imaging (MRI), and ultrasound (US). Ultrasound offers an important alternative, especially in women with dense breast tissue, because it is non-invasive and easily accessible. However, the interpretation of ultrasound images usually depends on the experience of the radiologist, which increases subjectivity. In recent years, artificial intelligence and deep learning-based systems have been developed for the automatic classification of breast lesions from ultrasound images and are integrated into clinical applications as an auxiliary tool in early diagnosis processes. Thus, the development of artificial intelligence-based automatic detection and diagnosis methods for medical image analysis increases diagnostic accuracy and reduces clinical workload. The accuracy rate is constantly increased when the studies conducted in this field in recent years are examined, and new generation architectures such as Vision Transformer (ViT) are being developed.

### 1.1. Related Works

Many artificial intelligence-based models are used for automatic detection using cancer images. Different models are used for the detection of cancerous images, segmentation of cancerous areas, and determination of cancer type. In this study, cancer images were classified using the BUSI dataset. Studies developed using the BUSI dataset in the literature were investigated and their contributions were examined. Table 1 summarizes the current studies and methods in the literature using the BUSI dataset.

Table 1 summarizes current studies on classification and segmentation using the BUSI dataset and highlights two major trends: (i) a transition from CNN-based models to hybrid CNN–transformer and pure transformer architectures, and (ii) an increasing use of segmentation methods to support classification. Classification approaches are generally developed either for binary setups (cancer vs. normal) or for three-class setups (normal, benign, malignant). Most studies rely on deep or machine learning methods, where feature extraction is typically performed with pre-trained models and then classified using deep or machine learning classifiers. Hybrid strategies that combine multiple models are often employed to achieve higher accuracy, and more recent approaches, such as the vision transformer, divide images into patches to capture long-range dependencies. Segmentation-based methods have also become prominent, supported by lesion masks available in the BUSI dataset, with architectures such as U-Net++, EfficientUNet, Attention-driven U-Net, and CMU-Net being widely used. While binary classification setups frequently achieve very high accuracies, the three-class scenario remains more challenging and requires ROI-focused preprocessing, stronger regularization, and class balancing. ROI-based approaches are particularly critical as they enable the model to focus on diagnostically relevant lesion regions while reducing the influence of noisy background tissue, thereby improving both accuracy and clinical interpretability. Over the past five years, transformer-based models, hybrid architectures, and optimized U-Net derivatives have increasingly dominated BUSI-related research. These developments formed the motivation for our proposed method, which explicitly combines ROI cropping to suppress background noise, Vision Transformer architectures to capture global contextual information, and focal loss to alleviate the inherent class imbalance in the BUSI dataset.

### 1.2. Contribution and Novelty

The main contributions of this study to the literature can be summarized as follows.

- ROI-Based Transformer Classification: Lesion regions (ROIs) were automatically extracted with mask files based on expert annotations, which were directly input to the model. Thus, only meaningful regions were classified.
- The use of vision transformer (ViT) models over traditional CNN-based approaches to capture global dependencies in breast ultrasound images, which are often overlooked by local receptive field structures in CNNs.
- A hybrid architecture combining ViT and a custom-designed MLP head, which enhances classification capability by capturing both global and non-linear patterns.
- The integration of an ensemble of ViT variants (ViT-Base, DeiT, and ViT-Small), which boosts robustness and generalization across different folds.
- The use of focal loss with a customized α coefficient to mitigate class imbalance—especially relevant due to the natural distribution of benign, malignant, and normal cases in the BUSI dataset.
- Advanced data augmentation techniques (CutMix, ElasticTransform) and test-time augmentation (TTA) strategies to prevent overfitting and improve generalization.
- Comprehensive Evaluation: The model was tested with 10-fold stratified cross-validation; many metrics, such as accuracy, precision, recall, F1-score, specificity, and AUC, were reported in detail on a class basis.
- High Applicability: High recall and AUC values were obtained for the clinically critical class malignant, demonstrating a reliable decision support potential for early diagnosis.

With these aspects, the study aims to fill the gap in the literature by addressing the applicability of ViT-based architectures in medical image classification in an ROI-aware and ensemble structure.

## 2. Materials and Methods

**Figure 1:**
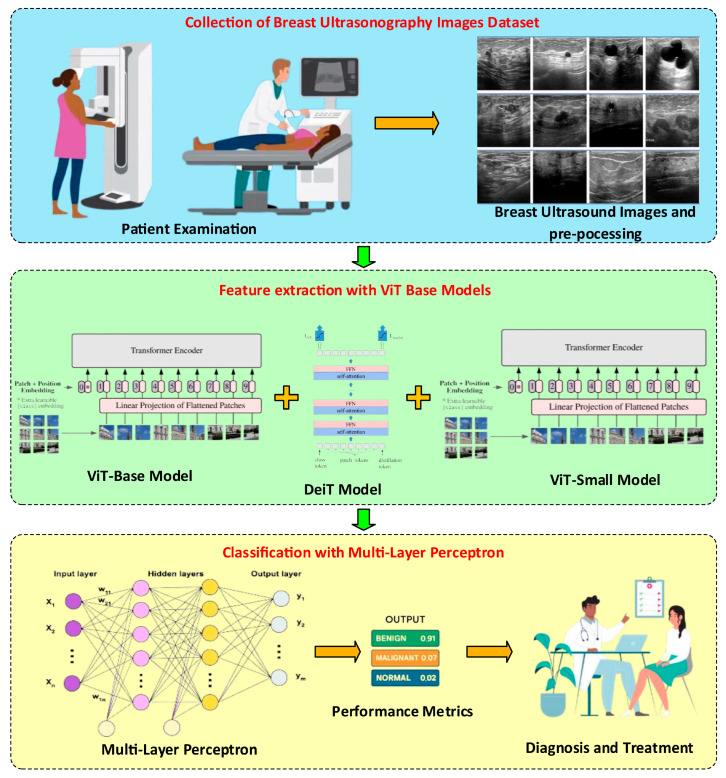
General block diagram of the proposed method.

In this study, a new classification approach based on mask-based ROI segmentation and ViT architecture is proposed to distinguish benign, malignant, and normal tissues in breast ultrasound images. The lesion region was extracted from each ultrasound image using expert annotation-based mask images. The obtained ROIs are enriched with various data augmentation techniques and given as input to pre-trained vision transformer (ViT) models. The representations obtained from the Transformer are classified using a multilayer MLP header structure. In addition, the results obtained from three different ViT models are combined with the ensemble method to improve the overall prediction performance. Focal loss and CutMix regularization strategies are applied to address class imbalance. The proposed method was tested with a 10-fold cross-validation, and its reliability was verified. The details of this approach are presented under headings in this section. Figure 1 summarizes the general architecture of the proposed method.

Figure 1 shows that the proposed method comprises three basic steps. In the first step, we investigated the BUSI dataset. Image preprocessing was performed on the BUSI dataset images. In the image preprocessing steps, the lesion region (ROI) was automatically extracted using the method. The purpose of determining and cropping the lesion regions and cropping them from the main image is to ensure that the model is trained on the real image. In particular, it provides feature extraction on lesion images instead of the details in the non-lesion regions. The number of images was then increased using general image augmentation algorithms. After the preprocessing step, feature extraction was performed on the normal, benign, and malignant images obtained. ViT-based (ViT_base, DeiT, and ViT_small) models were used for feature extraction. Due to the different structures of these three models, three different feature extractions were performed, and the obtained features were combined and used for classification. The multilayer perceptron model was used for the classification process.

### 2.1. BUSI Dataset Features

In this study, the BUSI breast ultrasound images dataset published by Al-Dhabyani et al. and made publicly available [3] was used. The dataset consists of **780** ultrasound images belonging to three different classes: **benign, malignant, and normal** (Table 2). The expert-created lesion area is shown in the binary mask files for each image. Because no lesion exists in the images belonging to the “Normal” class, there is no mask file for this class. This section may be divided by subheadings. It should provide a concise and precise description of the experimental results, their interpretation, as well as the experimental conclusions that can be drawn.

**Figure 2:**
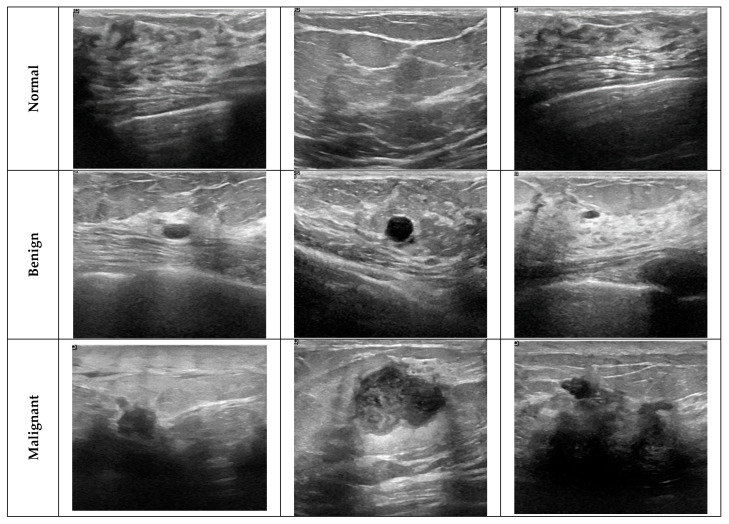
Sample images from the BUSI dataset.

The images are in png format and their resolutions vary. Mask files show each lesion area in binary format and are indicated with the _mask extension in the file names. These masks are used to determine the tumor area, allowing only meaningful (lesion-containing) regions to be included in the model input. This approach is important in terms of focusing the model’s attention only on relevant regions during the learning process. Sample images from the BUSI dataset are given in Figure 2.

### 2.2. ROI Segmentation and Image Preprocessing

For each ultrasound image belonging to the **benign and malignant** classes in the dataset, **the lesion region (ROI)** was automatically extracted using matching mask files. In this process, if multiple mask files corresponding to each image were used, these masks were combined by finding the maximum value pixel-by-pixel. The resulting combined mask defines the region where the tumor area. **The active pixel coordinates** of the obtained mask (i.e., pixels with values different from zero) were determined, and **a 5% horizontal and vertical buffer margin** was added around the area where the lesion was located, thus taking into account the tissues around the lesion. The following equation illustrates the establishment of the ROI boundaries were established in Equation (1).

(1) $$\begin{array}{ll} & x_{1}=max\left(0,x_{min}-w_{pad}\right)\\ & x_{2}=min\left(W,x_{max}+w_{pad}\right)\\ & y_{1}=max\left(0,y_{min}-h_{pad}\right)\\ & y_{2}=min\left(H,y_{max}+h_{pad}\right) \end{array}$$

Here (x_min_,x_max_) and (y_min_,y_max_) represent the boundaries of the masked lesion region, wpad, and hpad represent the buffer ratios calculated over the image width and height, respectively. Using these boundaries, only the region of interest (ROI) was cropped from the images, ensuring that the model learned only from tumor-containing tissues. This process both increases the generalization ability of the model and reduces the effect of noisy background data. After the ROI cropping process, all images were resized to 224 × 224 for model compatibility. During the normalization process, the transformation was made as in Equation (2) so that the mean μ = 0.5 and the standard deviation σ = 0.5 for the RGB channels:

(2) $$I_{norm}=\frac{I-\mu }{\sigma }$$

**Figure 3:**
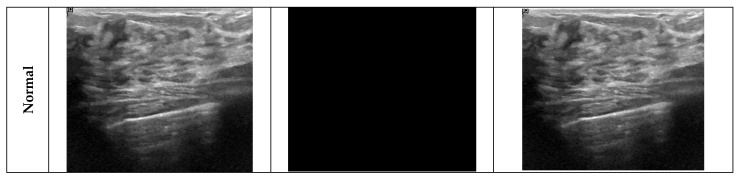

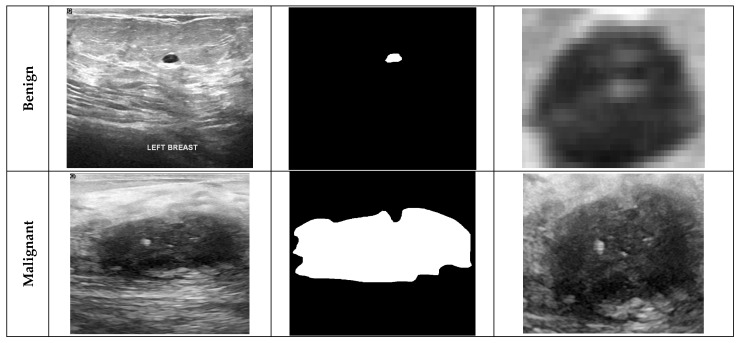
Sample images from the ROI segmentation process.

These preprocessing steps ensure that the data are processed more consistently and decisively by the model. This entire process was performed automatically during data loading, without any manual intervention. It was embedded in the custom BUSI Dataset PyTorch 2.5.0 class, and applies the same logic consistently for all images during both training and evaluation. This automated pipeline ensured that the model focuses only on the relevant lesion region, improving convergence and reducing background noise. Sample images from the ROI segmentation process are given in Figure 3.

### 2.3. Data Augmentation Techniques

To enhance the generalization capabilities of deep learning models and mitigate the risk of overfitting during the training process, **multiple data augmentation techniques** were applied to the training images. These methods were developed to modify the spatial arrangement of images (location, size, direction) and their photometric properties (luminance, sharpness, defocus). Thus, the model was made more robust to variations in real-world conditions. All augmentation operations were implemented using the **Albumentations** library, which is renowned for its powerful and flexible structure. During the training process, each image was subjected to the transformations shown in Table 3.

### 2.4. Vision Transformer-Based Feature Extraction

In this study, the vision transformer (ViT) architecture is used as an image-based classification model. Unlike traditional convolutional neural networks (CNNs), ViT divides images into small fixed-sized patches and feeds each one to a Transformer in turn. The Transformer architecture is notable for its ability to learn **global context relationships** between all patches and shows strong performance, especially in complex structures, such as medical images. The basic steps of the model can be summarized as follows:

Patch generation: The input image is of size $I\in R^{H\times W\times C}$ and is divided into small pieces of size **P × P**. The number of each patch is calculated in Equation (3).

(3) $$N=\frac{HW}{P^{2}}$$

Flattening and Embedding: Each patch is converted to a fixed-size vector using the linear layer and Equation (4):

(4) $$x_{p}=E\cdot \mathrm{Flatten}\;\left(p_{i}\right)$$

Here, E is the embedding weight matrix and represents the (pi)th patch.

Positional Embedding: The transformers lack position information; therefore, the fixed or learnable **positional embedding vectors** are summed with P (Equation (5)):

(5) $$z_{0}^{i}=x_{p}^{i}+P^{i}$$

Transformer Encoder Layers: Input vectors are passed through blocks consisting of multi-head self-attention (MHSA) and feedforward network layers (Equation (6)):

(6) $$\begin{array}{c} z_{l+1}=MHSA\left(LN\left(z_{l}\right)\right)+z_{l}\\ z_{l+1}=MLP\left(LN\left(z_{l+1}\right)\right)+z_{l+1} \end{array}$$

Here, LN represents the layer normalization process and MLP represents the two-layer fully connected network.

Classification Vector (CLS token): The [CLS] vector, which is specially placed at the end of the transformer, is used to represent the entire image. This vector is transferred to the classification head (MLP).

Vision transformer (ViT) is a deep learning architecture used in the field of image processing and is generally used in image classification [26]. Transformer architectures, which were first used in the field of natural language processing, have been adapted to the field of image processing thanks to ViT. The purpose of this model is to divide the image into pieces. It usually divides the 224 × 224 image into small squares of 16 × 16. Each divided small piece is flattened and turned into a vector. Then, positional encoding is added for the location information in the image. Thus, feature extraction is performed on the image.

**Figure 4:**
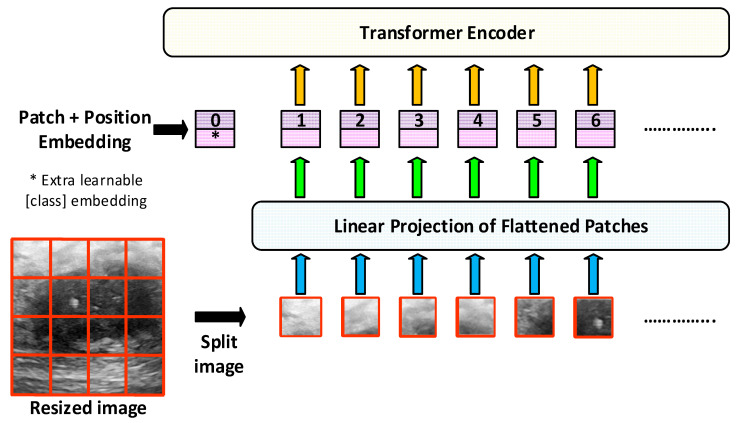
General architecture of the ViT model.

In this study, pre-trained ViT models such as vit_base_patch16_224, deit_base_distilled_patch16_224, and vit_small_patch16_224 are used. The general architecture of the ViT model is shown in Figure 4.

In this study, the base and small types of the basic ViT model given in Figure 3 are used. ViT-Small and ViT-Base models generally have the same vision transformer (ViT) architecture. However, they differ in terms of the parameter size, number of layers, and computational power. The ViT-Base model is preferred for deep analysis and strong classification performance in large datasets, where higher accuracy is required [26,27,28,29,30]. The ViT-Small model is used for rapid prototyping, resource-limited environments, and developing practical applications in smaller datasets [26]. Table 4 summarizes the general features of ViT-Base and ViT-Small models used in this study.

**Figure 5:**
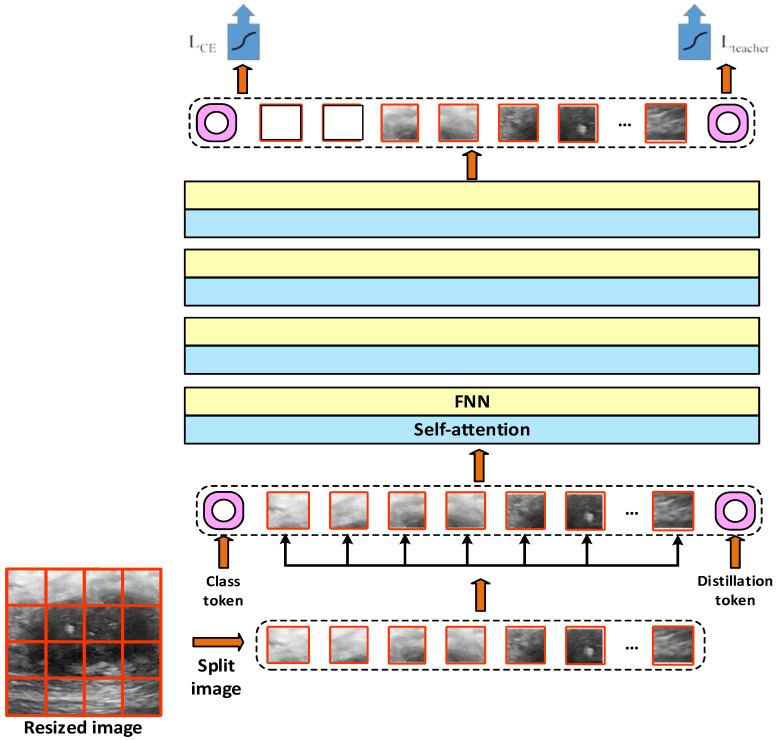
General architecture of the DeiT model [32].

In addition to the features obtained with ViT-Base and ViT-Small, we performed feature extraction using the DeiT (data-efficient image transformer) model. DeiT is similar to the classical vision transformer (ViT) architecture. However, it is optimized for less data and higher accuracy [31,32,33,34,35,36]. The general architecture of the DeiT model is presented in Figure 5 [32].

Figure 4 shows that according to the DeiT model, the images are arranged according to a size of 224 × 224. These fixed-size images are divided into small pieces of 16 × 16 size. Each patch is flattened and converted to a fixed-size vector. The class token and the DeiT-specific distillation token are also included. The position information is added to the patch vectors and tokens. It is passed through multilayer self-attention and feedforward network (FNN) blocks. Classification is performed using the Class and Distillation token. Information from both the image and teaching model is blended thanks to the distillation token. The outputs of these models are then transferred to the defined multilayer perceptron (MLP) classifier.

### 2.5. Multi-Layer Perceptron (MLP) Classifier

Although the Transformer architecture is quite successful in extracting image representations, a final layer structure is usually needed to adapt these representations to the classification task. In this study, a three-class classifier is designed by adding **a multilayer perceptron** (MLP) header structure to the end of the vision transformer (ViT) model. This MLP header takes the [CLS] vector obtained from the transformer as the input and produces the final class predictions. The layer structure of the MLP classifier header is listed in Table 5.

The general structure of the model can be represented mathematically as in Equation (7):

(7) $$ŷ=\mathrm{Softmax}\left(W_{2}\cdot \mathrm{Dropout}\left(\mathrm{ReLU}\left(W_{1}\cdot \mathrm{Dropout}(\mathrm{LayerNorm}(x))+b_{1}\right)\right)+b_{2}\right)$$

Here, x represents the transformer output vector, **W1,W2** weight matrices, **b1,b2** represent **bias** terms. Thanks to this structure, high-dimensional representations of the transformer are classified through nonlinear transformations, allowing more precise distinctions to be made between different types of lesions.

### 2.6. Loss Function with Focal Loss

One of the most important difficulties frequently encountered in medical image classification problems is **class imbalance**. In the BUSI dataset, there is a significant difference in the number of samples between the classes benign (437 samples), malignant (210 samples), and normal (133 samples). This situation may cause the model to be biased towards the majority class. In order to reduce this problem, **focal loss** is used instead of the traditional cross-entropy loss in this study. Focal loss encourages the model to learn these classes by increasing the weight of the samples belonging to rare classes in the loss function. Its basic formula is given in Equation (8).

(8) $$L_{\mathrm{focal}\;}=-\alpha_{t}{\left(1-p_{t}\right)}^{\gamma }log\left(p_{t}\right)$$

Here, pt is the probability that the model predicts the correct class. αt is the class-weight coefficient used to balance class imbalance. γ is the focusing parameter that reduces the contribution of correctly classified examples. When γ > 0, the model learns more about difficult examples. In this study, α = [1.0, 2.0, 1.0] is selected as the class weights (the malignant class is weighted twice as much), and γ = 2 is used as the focusing parameter. Thanks to this structure, the model can effectively recognize examples from minority classes without overfitting in the majority classes. Focal loss plays a critical role in improving performance, especially in the distinction between benign and malignant classes.

### 2.7. Editing with CutMix (Regularization)

In order to reduce overfitting in deep learning models and make the decision boundaries of the model more generalizable, a data augmentation-based regularization method called CutMix was applied in this study. The **CutMix** method combines two different images and performs a mixture at both the image and label level, allowing the model to learn more flexible representations. The CutMix method steps;

1. Two images (xA and xB) were randomly selected from the training samples.
2. A random rectangular region is determined from these images.
3. The pixels xB in the same region are pasted onto the selected region xA (Equation (9)):

(9) $$\tilde{x}=M⊙x_{A}+(1-M)⊙x_{B}$$

Here, M represents the binary mask matrix and ⊙ represents element-wise multiplication.

4. Similarly, the target labels are also shuffled according to the area covered by the region (Equation (10)):

(10) $$\tilde{y}=\lambda y_{A}+(1-\lambda )y_{B}$$

Here, λ∈[0, 1] denotes the area ratio of the pasted region in the image and is usually sampled by the **Beta distribution** (e.g., Beta(α,α). In this study, α = 1.0 is chosen. Thanks to the CutMix application, the model learns not only from complementary examples for each class but also from mixed examples, **making the decision boundaries smoother and more generalizable**. This increases the learning capacity of the model, especially in classes with low sample numbers.

### 2.8. Training Strategy and Cross Validation

In order to evaluate the reliability of the model and to use the limited size of the dataset more efficiently, **the Stratified k-Fold Cross Validation** method was applied in this study. This method divides the entire dataset into kkk subsets. One subset is used as validation data for each fold, and the remaining k − 1 subsets are used as training data. In addition, thanks to the **stratified** approach preserves the distribution of classes within each fold according to the ratios in the original dataset. The parameters used in the training process are shown in Table 6.

### 2.9. Ensemble Modeling Approach

In order to increase the classification performance of deep learning-based models and to strengthen model generalization, the **ensemble modeling** approach is adopted in this study. Ensemble learning combines the predictions of multiple models to form a single decision mechanism, thus reducing the impact of individual errors while benefiting from the strengths of each model. In this study, **the average ensemble** is created by combining the outputs of the three different **vision transformer** (ViT)-based models listed below (vit_base_patch16_224, vit_small_patch16_224, deit_base_distilled_patch16_224). Each model was trained on the same training data and the prediction probabilities (softmax scores) for the input image were obtained during validation. Then, the outputs of these models are specified in Equation (11).

(11) $${ŷ}_{\mathrm{ensemble}\;}=\frac{1}{N}\sum_{i=1}^{N}\;{ŷ}_{i}$$

Here ${ŷ}_{i}$ represents the prediction probability vector of the i-th model, N represents the total number of models (3 in this study),$\;{ŷ}_{\mathrm{ensemble}}$ represents the final prediction. The average prediction vector obtained is then assigned to the class with the highest probability using the **argmax** operation, and the final decision is made. Thanks to this method, the learning differences of each model are balanced, the over-learning effect caused by random variations is reduced, and the discrimination performance between classes is improved. The application of the ensemble strategy results in higher accuracy and F1-score than single models. These results demonstrate the effectiveness of ensemble modeling in medical image classification applications.

### 2.10. Performance Evaluation Metrics

In addition, to combat the **class imbalance** problem, a **weighted random sampler** was used during training, and samples belonging to minority classes were presented to the model more frequently during training. The sampling weights of each sample were inversely proportional to the class frequencies. The predictions and accuracy values obtained at the end of each fold were stored, and the average of the overall performance was calculated; thus, the generalizability of the model was evaluated in a statistically reliable manner.

## 3. Results

In this study, a comprehensive set of augmentation techniques was applied during training to improve the model’s generalization ability and to reduce the risk of overfitting. Using the Albumentations library, we implemented horizontal and vertical flipping, brightness and contrast adjustment, shift-scale-rotation, elastic transformation, Gaussian blur, and coarse dropout, each with carefully constrained parameter ranges to prevent unrealistic distortions while still introducing clinically meaningful variability. In addition to these transformations, CutMix was employed to generate mixed samples that smooth decision boundaries and improve minority-class learning, while test-time augmentation (TTA) was used to reduce prediction variance during inference. To quantify the effect of these augmentations, we conducted a comparative experiment against a baseline model trained on the original dataset without any augmentation. The results, summarized in Table 7, clearly demonstrate the effectiveness of the augmentation strategy: accuracy increased from 87.92% to 94.03%, macro-averaged F1-score improved from 0.8614 to 0.9300, and recall values also showed substantial gains across classes. These findings highlight that augmentation techniques such as CutMix, elastic transformation, and brightness–rotation not only act as a safeguard against overfitting but also significantly enhance the robustness and generalization capability of the proposed model in the challenging three-class classification of breast ultrasound images.

Detailed evaluation results of the proposed ViT + MLP + Ensemble-based classification model on the BUSI dataset for three classes (benign, malignant, normal) are presented in Table 8. The accuracy, precision, recall, specificity, and F1-score metrics calculated for each class comprehensively reveal the success of the model on a class basis. Additionally, training time and parameter size are reported, indicating that the model converges efficiently (~810 s) despite its relatively high capacity (~86 million parameters).

**Figure 6:**
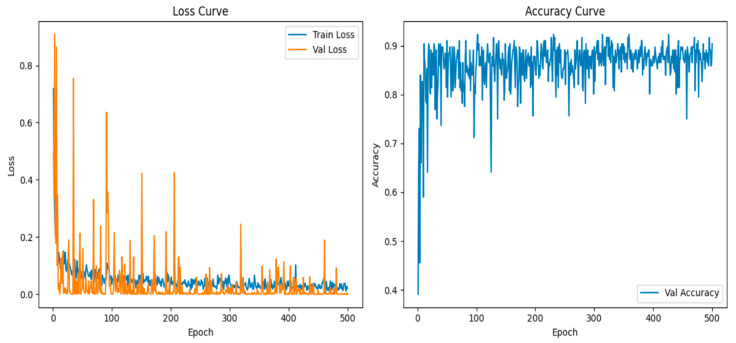
Accuracy and loss plots of the proposed method for train dataset.

The results obtained for the **benign** class show that the model recognizes images belonging to this class with high accuracy. The high **precision value of 0.9620** shows that nearly all the samples predicted as benign actually belong to the correct class. On the other hand, the **recall value of 0.8636** shows that the model may miss some benign samples (false negatives). Nevertheless, the **F1-score of 0.9102** indicates a balanced overall performance. **The malignant class** is of particular importance as it is the most critical class from a clinical perspective. **The recall value** obtained for this class is quite high at 0.9286, indicating that the model has a high rate of correctly recognizing malignant cases. However, **the precision** value is relatively low at **0.7647**; in other words, the model may occasionally incorrectly predict benign samples as malignant (false positive). This situation is also reflected in the relatively **low specificity value of 0.8947**. However, the **F1-score of 0.8387** indicates a successful overall recall performance. The performance of the model for **the normal class** is close to perfect. All metrics were calculated as **1.0000**, indicating that the model correctly recognized all samples in the normal class and did not cause any confusion. This shows that the proposed method can reliably distinguish healthy tissues from other tissues. In general, the proposed method produced extremely successful results in the benign and normal classes and was successful in capturing clinically critical samples by maintaining high sensitivity in the malignant class. These results strongly support the applicability of the proposed method to medical decision support systems. Figure 6 (loss curve and accuracy curve) visualizes how **the loss and accuracy** values of the proposed model in the training process change with the number of epochs. When **the loss curve** is examined, both training and validation losses generally show a decreasing trend, and the model makes fewer errors over time. In particular, the sudden jumps observed in validation loss are quite natural in cases such as medical imaging, where the data distribution is complex and the number of classes is unbalanced.

**Figure 7:**
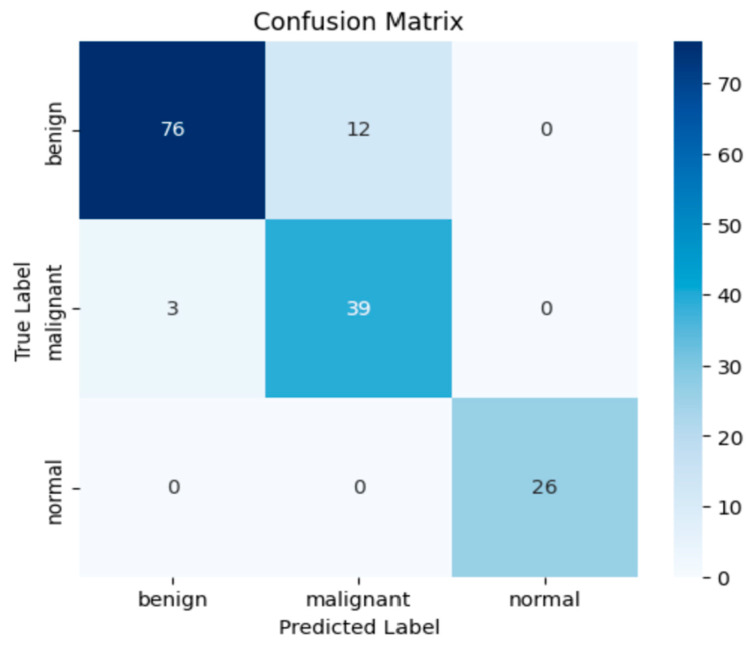
Confusion matrix.

Despite these fluctuations, the low level of general loss shows that the model performs a balanced learning process away from the **overfitting tendency. The Validation Accuracy Curve** shows that the model quickly reaches high accuracy levels after the first few epochs and largely maintains this level during the training period. The fact that the validation accuracy is fixed at 90% levels in the training conducted for 500 epochs shows that the model has learned **a stable and generalizable structure**. Accuracy fluctuations observed in early epochs indicate local errors that may arise from imbalance, especially in minority classes. These curves show that the model **has high success** on both training and validation data, avoids overfitting, and develops a robust generalization capacity. Figure 7 presents the confusion matrix results.

**Figure 8:**
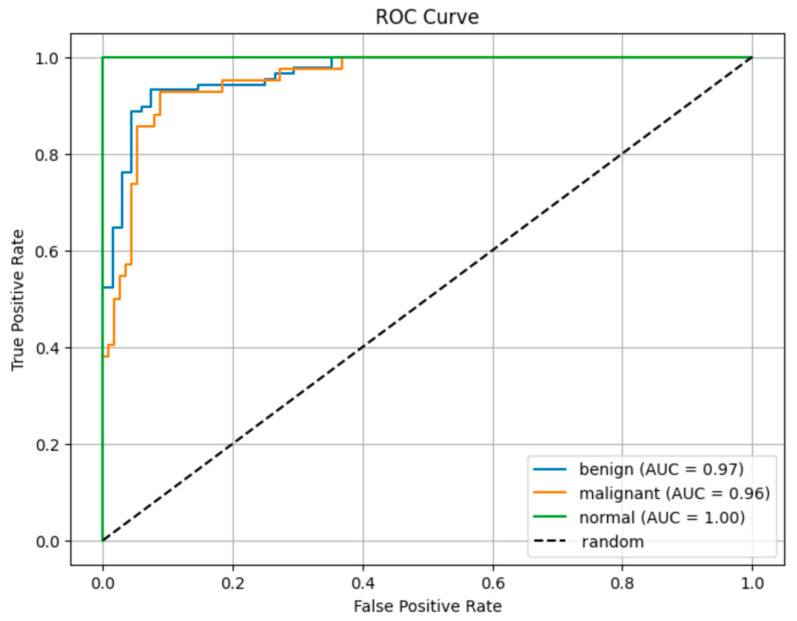
ROC Curve.

The confusion matrix presented in Figure 6 provides a detailed insight into the classification performance of the proposed model. Out of 88 benign samples, 76 were correctly classified (accuracy: 86.4%), while 12 were misclassified as malignant. For the malignant class, the model achieved a correct classification rate of 92.9%, with 39 out of 42 samples accurately predicted and only 3 misclassified as benign. Most notably, the normal class exhibited perfect classification performance, with all 26 samples correctly identified, yielding a class accuracy of 100%. These results underline the model’s strong sensitivity and precision, particularly in detecting malignant lesions, which are clinically critical. The overall confusion matrix supports the model’s high discriminative power and robust generalization across all classes. Figure 8 shows the ROC curve results.

The ROC curves in Figure 7 show the class discrimination performance of the proposed model. A comparison of AUC values based on ROC curves reveals that the proposed model has high discrimination power among all classes. **The AUC value of 0.96** obtained in the malignant class shows that the model can successfully recognize this clinically critical class with high success. **The AUC value of 1.00** for the normal class proves that the model distinguishes the samples in this class without error. The AUC of 0.97 obtained in the benign class shows that the model can successfully distinguish benign cases from malignant ones. The fact that all AUC values are above 0.95 reveals that the model offers **a strong general discrimination capacity despite the unbalanced data distribution.**

## 4. Discussion

In this study, the proposed model approach based on ViT, which was developed to classify benign, malignant, and normal tissues from breast ultrasound images, is supported by mask-based ROI segmentation, advanced data augmentation, focal loss, CutMix, and ensemble strategies.

To evaluate the contribution of the ensemble strategy, each individual ViT-based model was also trained and tested independently using the same experimental setup. Table 9 compares the performance metrics of ViT-Base, DeiT-Base, and ViT-Small with the proposed ensemble model. While all standalone ViT architectures achieved relatively high classification accuracy (above 90%), the ensemble approach outperformed all individual models, achieving 94.03% accuracy, 0.94 precision, 0.93 recall, and an F1-score of 0.935. These results validate that combining complementary transformer architectures through an ensemble strategy significantly enhances model robustness and generalizability.

To comprehensively evaluate the performance of the proposed ViT + MLP ensemble architecture, we compared it with several widely adopted deep learning models in medical image classification, including ResNet50, DenseNet121, VGG19, and Xception (Table 10). The results showed that our method achieved the highest overall accuracy (94.03%) and the highest macro-averaged F1-score (0.935) among all models evaluated. While DenseNet121 and Xception performed competitively in terms of F1-score and parameter efficiency, the ViT-based ensemble model demonstrated clear superiority in both global feature extraction and multi-class discrimination, confirming the advantage of incorporating Transformer-based architectures. In addition to outperforming CNN-based models, our ensemble design, despite its relatively higher parameter size (~258 million), provides a favorable trade-off between accuracy and computational complexity, as it significantly improves robustness and generalization across the three-class setup. Class-wise evaluations further highlight the strengths and limitations of the model: for the malignant class, recall was high at 0.9286, ensuring that clinically critical malignant lesions were rarely missed, but precision was relatively lower at 0.7647, indicating an increased false positive rate where some benign lesions were misclassified as malignant. In contrast, the normal class achieved perfect scores across all metrics (accuracy, precision, recall, F1 = 1.0000), and ROC/AUC values further validated the discriminative power of the model with 0.97 (benign), 0.96 (malignant), and 1.00 (normal). These strong results are attributed to the synergy of ROI cropping, which minimizes background noise; transformer-based attention, which captures long-range contextual dependencies; and focal loss with weighted sampling, which addresses the imbalance between benign, malignant, and normal cases. Nevertheless, several shortcomings remain: (i) the recall–precision trade-off in the malignant class, (ii) dependence on expert-provided ROI masks that may limit deployment in mask-free clinical workflows, (iii) the relatively large parameter size of the ensemble model, (iv) reliance on a single-center dataset with inherent class imbalance, and (v) the absence of patient-level aggregation strategies that could further reduce false positives. Future work will focus on addressing these limitations through multi-center validations, weakly supervised methods that remove ROI dependency, lightweight transformer architectures for clinical feasibility, and improved calibration and patient-level decision strategies.

The experimental results show that the proposed model exhibits high accuracy and discriminative performance in all three classes. The high sensitivity (recall: 0.9286) obtained in the malignant class shows that the model can successfully detect clinically critical malignant lesions to a large extent. However, the lower precision value (0.7647) indicates that some benign samples were incorrectly classified as malignant. Although this situation potentially increases the false positive rate of the model, it offers a significant advantage in terms of not missing malignant lesions. The focal loss function and class-weighted sampling play an important role in this result. Obtaining 100% accuracy and AUC (1.00) in the normal class shows that the model is extremely successful in distinguishing healthy tissues from lesions. This is quite valuable in terms of both the low error rate and reliability of decision support systems. The success of the model can be associated with the Vision Transformer architecture’s ability to learn global context relationships in medical images more effectively than CNN-based approaches. In addition, combining ViT models with the ensemble (multiplexing) method increased the generalization capacity of the model and provided consistent performance on validation sets. When the curve analyses (loss, accuracy) were examined, it was determined that the model performed stable learning during the training process and that there was no over-learning tendency. However, this study has some limitations. First, the fact that the dataset was single-center and had a limited number of samples may not fully reflect the performance of the developed model on real-world data. In addition, the imbalance in the class distribution (benign > malignant > normal) made learning minority classes difficult for the model. Future studies with multi-center, larger, and balanced data sets will allow a more reliable test of the external validity of the model. This study has shown that ViT-based approaches provide promising results in medical image classification. The developed model is a strong candidate for clinical decision support systems owing to its high accuracy and reliable classification performance. The comparison of the performance of the proposed method with studies in the literature is listed in Table 11.

Recently, various deep learning-based approaches have been used in the classification of breast ultrasound images. Table 11 shows that the success rates of the methods in the literature vary depending on the model architecture used, data augmentation strategies, and the addition of innovative components. In the studies conducted by Walid Al-Dhabyani et al. [7] on the BUSI dataset, the classical CNN architecture AlexNet was used and the effect of data augmentation methods was investigated. The highest accuracy reached 94% with the integration of DAGAN augmentation and transfer learning were integrated. However, the model structure remained at the basic CNN level, and more modern Transformer-based architectures were not evaluated. Jabeen et al. [1] tested different deep learning architectures together with traditional augmentation methods and reported the highest success rate in the literature as 99.1% with the combination of DarkNet-53 + RDE + RGWt + Fusion. However, the model complexity in this study was quite high, and region-based analysis or a transformer architecture was not used. A comprehensive study conducted by Pa [6] compared both CNN-based (AlexNet, VGG16, ResNet series, EfficientNet) and transformer-based (Swin transformer, vision transformer) models. The use of the vision transformer achieved 88.6% accuracy. Techniques, such as ensemble learning and focal loss, that reduce class imbalance were also not used. Gheflati et al. [2] calculated success rates of 82% and 83% by comparing the ViT-B/32 and ResNet models. Although this result shows the advantages of the ViT architecture, we did not try smaller ViT variants (e.g., ViT-Small), or strong optimization techniques were not tried. Liu et al. [10] achieved 97.18% accuracy with a deep neural network (DNN) based structure, but they did not include transformer architectures and regional images. Ali et al. [11] reported 90% accuracy with the ResNet50, DenseNet121, and ensemble meta-model approaches. However, the potential of the transformer structure was not evaluated in this study. Xiong et al. [13] achieved 88.6% accuracy using UNet + ResNet combinations and SIFT-based methods. Although image segmentation integration is provided, transformer models are excluded.

Newer studies also demonstrate strong performance on BUSInusing diverse architectures and strategies. CNN-based methods with VGG16 backbones [15], customized AlexNet variants [16], and EfficientNet-B7 with explainable AI [20] have reported accuracies exceeding 96%, with some reaching over 99%. Hybrid CNN–transformer approaches [17] and advanced feature fusion techniques [14] have also demonstrated strong performance, while other works explored alternative paradigms such as graph convolutional networks [18] and meta-learning frameworks [19].

In comparison, our proposed method (ROI extraction + ViT + MLP + Ensemble + focal loss) achieves 94.03% accuracy while addressing key limitations observed in many high-performing CNN-only models. By integrating transformer-based feature extraction, the model captures long-range dependencies and richer contextual information from lesion-focused regions, improving generalization across varied cases. The ensemble structure enhances robustness by combining complementary decision boundaries, while focal loss mitigates class imbalance—critical given the uneven class distribution in BUSI. Moreover, the ROI-based preprocessing ensures that the network focuses on diagnostically relevant regions, reducing background noise and improving interpretability. This balanced combination of accuracy, robustness, and clinical relevance positions our approach as a strong candidate for real-world deployment, with the added flexibility to incorporate explainable AI frameworks and multi-modal data in future work.

## 5. Limitations and Future Work

Although the ViT-based model proposed in this study has shown high classification success on the BUSI breast ultrasound image dataset, some limitations should be considered. First, the dataset used consists of a single-center and relatively small sample. This may limit the generalizability of the model on images obtained using different patient groups and devices. In addition, the significant imbalance in the class distribution (dominance of the benign class) has led to the risk that the model may not adequately represent minority classes (malignant, normal). In addition, all data used in this study are limited to images with labeled and mask files. Mask information is often not available in real clinical environments; therefore, it is important to develop models that can directly classify full images without masks. In future studies, the model will be tested on multi-center and larger datasets. In addition, the ability to adapt to different ultrasound devices can be increased with transfer learning. In addition, in order to increase the explainability of the model, the decision-making process can be made more transparent with **attention maps, Grad-CAM, or transformer-based visualization techniques**. The development of mobile-compatible, low-latency, lightweight ViT architectures to increase clinical applicability is also among the future work.

## 6. Conclusions

In this study, a ViT-based classification model was proposed, extensively tested, and evaluated on the BUSI breast ultrasound image dataset for three classes (benign, malignant, normal). Mask-based region of interest (ROI) cropping, data augmentation, CutMix, focal loss, and ensemble strategies were used to support the model. Performance evaluations were performed with 10-fold cross-validation (stratified 10-fold CV) and measured using metrics such as accuracy, precision, sensitivity, specificity, F1-score, and AUC. In the benign class, the model exhibited high reliability performance with 96.2% precision and 86.3% recall. In the malignant class, a 92.9% recall was obtained, demonstrating that the model can detect malignant lesions with high sensitivity. In the normal class, 100% success was achieved in all metrics, and the model could clearly distinguish healthy tissues from others. According to the confusion matrix analysis, the model occasionally confused benign samples with malignant samples, but it successfully maintained its discrimination power between classes. ROC curves also supported this success, with **AUC = 0.97** and **AUC = 0.96** for benign and malignant classes, respectively, and **AUC = 1.00** for the normal class. Additionally, the validation accuracy curve observed throughout the training process shows that the model provides stable learning, whereas the validation loss curve shows that overlearning is not encountered. With the contribution of the ensemble structure, the generalization power of the model against different variations has increased, and stable success has been achieved, especially in minority classes.

## Figures and Tables

**Table 1:** Current studies and methods in the literature using BUSI dataset.

Classification/ Segmentation	Reference	Year	Method
Classification	Walid Al-Dhabyani et al. [7]	2019	TL-NASNet
	Jabeen et al. [1]	2022	DarkNet-53 + RDE + RGW + Fusion
	Pacal [6]	2022	Vision_transformer
	Gheflati et al. [2]	2022	ViT-B/32/ResNet
	Liu et al. [10]	2022	DNN
	Ali et al. [11]	2023	CNN
	Mishra et al. [12]	2020	Machine learning
	Xiong et al. [13]	2023	UNet + ResNet + SIFT
	Wei et al. [14]	2024	Multi-feature fusion multi-task (MFFMT)
	Afrifa et al. [15]	2023	CNN with VGG16
	Arooj et al. [16]	2022	Customized CNN-AlexNet
	Meng et al. [17]	2024	CNN and transformer models
	Montaha et al. [18]	2024	A graph convolutional network (GCN)
	Işık et al. [19]	2024	Prototypical networks and model agnostic meta-learning (MAML)
	Latha et al. [20]	2024	EfficientNet-B7 and Explainable AI
	Saini et al. [21]	2025	CNN
Segmentation	Sulaiman et al. [22]	2024	Attention-driven U-Net
	Zhang et al. [23]	2024	CMU-Net
	Almajalid et al. [24]	2018	U-Net
	Shang et al. [25]	2024	U-Net++
	Dar et al. [9]	2023	EfficientU-Net

**Table 2:** BUSI dataset image features [3].

Class	Number of Images	Mask Info
Benign	437	Yes
Malignant	210	Yes
Normal	133	No (without lesion)
**Total**	**780**	Mixed

**Table 3:** Data augmentation techniques used.

Conversion	Description	Implementation Probability (*p*)
Horizontal/Vertical Flip	Image rotation symmetrically about the axis	0.5
Brightness/Contrast Adjustment	Modifying the light areas	0.5
Scroll-Scale-Rotate	Spatial variations	0.5
Elastic Transform	Simulates texture distortions	0.3
Gaussian Blur	Defocus effect	0.2
Coarse Dropout	Random region deletion	0.5
Resize	Setting to 224 × 224 pixels	Always
Normalize	Per channel μ = 0.5\mu = 0.5μ = 0.5, σ = 0.5\sigma = 0.5σ = 0.5	Always

**Table 4:** General features of the ViT-Base and ViT-Small models used in this study.

Models Type	Patch Size	Number of Layers	Hidden Size	Heads	Parameters
**ViT-Base**	16 × 16	12	768	12	85 M
**ViT-Small**	16 × 16	8	512	8	22 M

**Table 5:** The layer structure of the MLP classifier.

Layer Number	Layer Type	Dimension Conversion	Description
1	Layer Normalization	R^D^→R^D^	Transformer output vector is normalized
2	Dropout (*p* = 0.3)	-	Random reset to prevent over-learning
3	Linear (Tam Bağlantı)	R^D^→R^512^	Transformation from input dimension to 512 dimension
4	ReLU Aktivasyon	-	Non-linearity is introduced
5	Dropout (*p* = 0.3)	-	Second regularization random reset
6	Linear (Çıkış Katmanı)	R^512^→R^3^	Output to three classes (benign, malignant, normal)
7	Softmax	R^3^→[0, 1]	Normalization of class probabilities

**Table 6:** Parameters used in the training process.

Component	Value /Method	Description
Cross Validation	10-fold stratified CV	Data is divided into 10 parts while preserving class distribution
Number of Epochs	50	Number of training iterations for each fold
Batch Size	16	Mini-batch size
Optimization Algorithm	AdamW	Adam optimization algorithm with weight decay
Learning Rate (η)	3 × 10^−4^	Initial learning rate
Weight Decay Coefficient (λ)	1 × 10^−4^	Regularization to reduce over-learning
Class Imbalance Measure	Weighted random sampler	Allows minority classes to be sampled more frequently
Sampling Weights (wi)	wi = 1/fci	Inversely proportional weight is given according to the frequency of each class
Training-Loss Function	Focal loss (γ = 2, α = [1.0, 2.0, 1.0])	Custom loss function robust against class imbalance
Regularization Techniques	Dropout, CutMix, data augmentation	Preventive strategies against over-learning
Test Time Augmentation (TTA)	3 × TTA	Average prediction from 3 augmented views of each test image
Fine-Tuning Strategy	Last block + normalization layers unfrozen	Only the last transformer block and normalization layers are updated
Data Augmentation	Resize, flip, brightness, rotation, blur, etc.	Advanced transformations applied during training

**Table 7:** Impact of data augmentation on model performance.

Setting	Accuracy (%)	Recall	Precision	F1-Score
Without Augmentation	87.92	0.8558	0.8625	0.8614
With Augmentation (Proposed)	94.03	0.9307	0.9325	0.9300

**Table 8:** Performance results of the proposed method.

Class	Accuracy	Precision	Recall	F1-Score
**Benign**	0.9038	0.9620	0.8636	0.9102
**Malignant**	0.9038	0.7647	0.9286	0.8387
**Normal**	1.0000	1.0000	1.0000	1.0000

**Table 9:** Performance comparison of individual ViT models and the proposed ensemble model.

Model	Accuracy (%)	Precision	Recall	F1-Score
ViT-Base Patch16-224	92.13	0.91	0.90	0.905
DeiT-Base Distilled Patch16-224	91.45	0.89	0.88	0.885
ViT-Small Patch16-224	90.72	0.88	0.87	0.875
Proposed Ensemble (Average)	94.03	0.94	0.93	0.935

**Table 10:** Comparison of different deep learning models in terms of performance and parameter size.

Model	Accuracy (%)	Precision	Recall	F1-Score	Parameters (M)	Training Time (min)
ResNet50	89.10	0.8765	0.8731	0.8743	25.6	11.2
DenseNet121	90.25	0.8875	0.8920	0.8901	8.0	9.8
VGG19	87.43	0.8603	0.8595	0.8582	143.7	13.1
Xception	91.67	0.9082	0.9038	0.9056	22.9	12.4
**Proposed Ensemble**	**94.03**	**0.9325**	**0.9307**	**0.9300**	**258.0**	**19.2**

**Table 11:** Comparison of the performance of the proposed method with the studies in the literature.

Reference	Dataset	Augmentation	Methods/Model	Accuracy (%)
Walid Al-Dhabyani et al. [7]	BUSI	No	CNN -AlexNet	58.0
			TL-NASNet	83.0
		Traditional	CNN -AlexNet	62.0
			TL-NASNet	85.0
		DAGAN	CNN -AlexNet	73.0
			TL-NASNet	91.0
		Traditional + DAGAN	CNN -AlexNet	78.0
			TL-NASNet	94.0
Jabeen et al. [1]	BUSI	Traditional	DarkNet-53 + RDE + RGW + Fusion	99.1
Pacal [6]	BUSI	Traditional	AlexNet	79.5
			VGG16	85.4
			VGG19	78.6
			GoogleNet	75.6
			ResNet18	82.6
			ResNet34	83.8
			ResNet50	83.1
			ResNet101	84.7
			EfficientNet	85.6
			Swin_Transformer	81.9
			Vision_Transformer	88.6
Gheflati et al. [2]	BUSI	Traditional	ViT-B/32	82.0
			ResNet	83.0
Liu et al. [10]	BUSI	No	DNN	97.18
Ali et al. [11]	BUSI	Traditional	Inception V3	83.0
			ResNet50	88.0
			DenseNet121	84.0
			Ensemble Meta-Model	90.0
Ziong et al. [13]	BUSI	Traditional	UNet + ResNet	81.9
			UNet + ResNet + SIFT	88.6
			Inception V3 + SIFT	87.9
		No	Inception V3 + SIFT	84.7
Wei et al. [14]	BUSI	Traditional	MFFMT	95.0
Afrifa1 et al. [15]	BUSI	Traditional	CNN with VGG16	96.10
Arooj et al. [16]	BUSI	No	Customized CNN-AlexNet	99.4
Meng et al. [17]	BUSI	Traditional	CNN and transformer models	98.72
Montaha et al. [18]	BUSI	No	A graph convolutional network (GCN)	91.03
Işık et al. [19]	BUSI	Traditional	Prototypical networks and model agnostic meta-learning (MAML)	88.9
Latha et al. [20]	BUSI	Traditional	EfficientNet-B7 and Explainable AI	99.18
Saini et al. [21]	BUSI	No	CNN	93.0
Our Method	BUSI	Traditional	ROI + ViT + MLP + Ensemble + FocalLoss	94.03

## Data Availability

The dataset used in this study, titled “The Breast Ultrasound Images (BUSI)”, is publicly available on Kaggle [19].

## Abbreviations

**CNN**	**Convolutional neural network**
RDE	Reformed differential evaluation
RGW	Reformed differential evaluation
TL	Transfer Learning
ViT	Vision_Transformer
DNN	Deep neural network
MLP	Multilayer perceptron
DeiT	Data-efficient image iransformer
ROI	Region of interest
AUC	Area under the curve
BUSI	The breast ultrasound images
